# Role of the leucine-rich repeat protein kinase 2 C-terminal tail in domain cross-talk

**DOI:** 10.1042/BCJ20230477

**Published:** 2024-02-19

**Authors:** Pallavi Kaila Sharma, Jui-Hung Weng, Jascha T. Manschwetus, Jian Wu, Wen Ma, Friedrich W. Herberg, Susan S. Taylor

**Affiliations:** 1 Department of Pharmacology, University of California, San Diego, La Jolla, CA 92093-0652, U.S.A.; 2 Department of Biochemistry, University of Kassel, Heinrich-Plett-Str. 40, 34132 Kassel, Hessen, Germany; 3 Department of Physics, University of Vermont, Burlington, Vermont; 4 Department of Chemistry and Biochemistry, University of California, San Diego, La Jolla, CA 92093-0652, U.S.A.

**Keywords:** C-terminal helix, GaMD simulation, LRRK2, Parkinson's disease, peptide array

## Abstract

Leucine-rich repeat protein kinase 2 (LRRK2) is a multi-domain protein encompassing two of biology's most critical molecular switches, a kinase and a GTPase, and mutations in LRRK2 are key players in the pathogenesis of Parkinson's disease (PD). The availability of multiple structures (full-length and truncated) has opened doors to explore intra-domain cross-talk in LRRK2. A helix extending from the WD40 domain and stably docking onto the kinase domain is common in all available structures. This C-terminal (Ct) helix is a hub of phosphorylation and organelle-localization motifs and thus serves as a multi-functional protein : protein interaction module. To examine its intra-domain interactions, we have recombinantly expressed a stable Ct motif (residues 2480–2527) and used peptide arrays to identify specific binding sites. We have identified a potential interaction site between the Ct helix and a loop in the CORB domain (CORB loop) using a combination of Gaussian accelerated molecular dynamics simulations and peptide arrays. This Ct-Motif contains two auto-phosphorylation sites (T2483 and T2524), and T2524 is a 14-3-3 binding site. The Ct helix, CORB loop, and the CORB-kinase linker together form a part of a dynamic ‘CAP’ that regulates the N-lobe of the kinase domain. We hypothesize that in inactive, full-length LRRK2, the Ct-helix will also mediate interactions with the N-terminal armadillo, ankyrin, and LRR domains (NTDs) and that binding of Rab substrates, PD mutations, or kinase inhibitors will unleash the NTDs.

## Introduction

**Figure 1.**
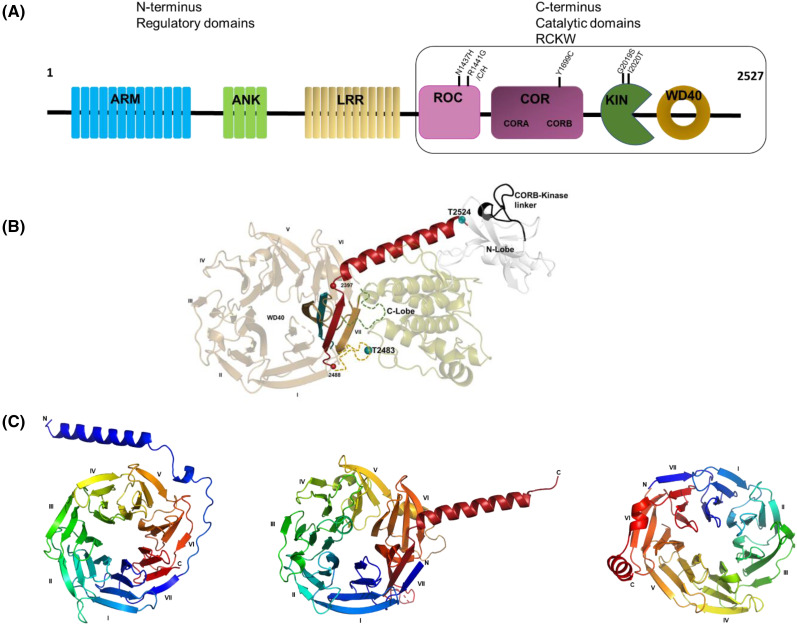
Domain organization of LRRK2. (**A**) Schematic representation of LRRK2 protein depicting the armadillo (ARM) repeats, followed by ankyrin (ANK) repeats, leucine-rich repeats (LRR), a ROC domain, a COR domain, a protein kinase domain, and the WD40 domain. The most common PD mutations (N1437H, R1441G/C/H, Y1699C, G2019S, I2020T) are marked in the catalytic half, RCKW. (**B**) Cartoon representation of RCKW (PDB: 6VNO) showing the C-terminal helix (Ct helix, in red) extending from the WD40 with the two predicted autophosphorylation sites, T2483 and T2524 seen as green spheres along with the COR kinase linker in black. (**C**) Cartoon representation of WD40 repeats showing the canonical seven-bladed structure folded into β-propeller architecture, on left is a WD40 domain with an extended N-terminal helix from a *Bos taurus* G protein (PDB ID: 1TBG), middle is the WD40 domain of LRRK2 showing the Ct helix from the cryo-EM structure of LRRK2 RCKW (PDB ID: 6VNO), right is a WD40 domain with a C-terminal helix from the S layer protein from *Geobacillus stearothermophilus* (4U1E).

Leucine-rich repeat protein kinase 2 (LRRK2), or dardarin, is a large multi-domain cytoplasmic protein mainly expressed in brain, lungs, macrophages, and kidney [1], and mutations in LRRK2 are one of the major causes of inherited and sporadic Parkinson's disease (PD). LRRK2 begins with 13 armadillo (ARM) repeats, followed by 7 ankyrin (ANK) repeats, 14 leucine-rich (LRR) repeats, a Ras-of-complex (ROC) domain, a C-terminal of Roc (COR) domain, a protein kinase domain, and seven WD40 repeats. It encompasses two of biology's most relevant molecular switches, a kinase and a GTPase, but its physiological role is still poorly understood. Based on the knowledge about functions of these domains, they can be divided into the N-terminal interaction domains (NTDs), including the ARM, ANK, and LRR repeats, and the C-terminal catalytic domains (CTDs), including the ROC, COR, Kinase, and WD40 (also referred to as the RCKW) (Figure 1A).

The most common mutations in LRRK2 contributing to the risk of PD are R1441C, R1441G, Y1699C, G2019S, and I2020T substitutions [2]. Some evidence for R1628P and G2385R is also available [1,2]. Of these mutations, five (i.e. N1437H, R1441C/G/H/S) are in the ROC domain, Y1699C is in the CORB domain, and G2019S and I2020T are in the kinase domain. Most of the mutations lead to an increase in the kinase activity of LRRK2, which is thought to correlate with the disease phenotype [3]. GTP binding to the ROC domain may also regulate kinase activity, stability, and localization [4]. This suggests an interdependence of the GTPase and kinase domains of LRRK2, and the cross-talk between these domains likely plays a major role in LRRK2's pathological function.

The recent availability of LRRK2 structures: crystal structure of the WD40 domain of LRRK2 [5], cryo-EM structures of the inactive RCKW [6], inactive full-length LRRK2 [7,8], and active full-length LRRK2 and Rab29 complexes in combination with AlphaFold [9] have helped the community to not only understand better the dynamics of activation and the function of LRRK2 but also opened doors to explore the physiological role of LRRK2.

We have previously demonstrated how the LRRK2 kinase domain conformation is regulated by its flanking domains using mutational analysis, Hydrogen-Deuterium eXchange Mass Spectrometry (HDX-MS), and Gaussian Accelerated Molecular Dynamics (GaMD) [10]. This has led to a fair amount of understanding of the kinase domain dynamics and the allosteric interactions leading to its different conformational states.

In the present study, we focus on one of the most overlooked features of LRRK2, the C-terminal helix (Ct helix, following the WD40 domain), which is stably docked onto the kinase domain of all the known LRRK2 structures (Figure 1B). Until now, its function and significance are not well explored. Previous studies suggest that it plays an essential role in kinase activity as its deletion leads to the abolishment of kinase activity [11]. Deletion of even the last three residues leads to a significant reduction in kinase activity [12]. A mass spectrometry-based cross-linking study showed that K2520 in the Ct helix cross-links to K773 in the ANK domain [13]. We have examined the Ct helix motif (residues 2480–2527) for its sequence and potential function using a combination of techniques: peptide arrays, HDX-MS, and GaMD. The sequence reveals this motif as a hub of (auto)phosphorylation sites (T2483 and T2524) [14,15], a putative PKA phosphorylation site (S2525), and a few intracellular localization motifs. We have recombinantly expressed and purified this motif in *Escherichia coli* to explore its interactions with other LRRK2 domains. Finally, we have shown by MD simulations that PD mutations in other domains appear to influence the Ct helix. There is a highly dynamic potential cross-talk between the C-terminal residues with the N-lobe of the kinase and the CORB domain. The carboxyl group of the C-terminal glutamic acid is in close proximity to many basic residues, and additional phosphates at T2524 or S2525 can contribute in more ways to domain cross-talk.

## Results

### Structural analysis of the WD40 domain

The WD40 repeat was first identified in the G_β_ subunit of heteromeric G proteins and cell-cycle protein CDC4 [16]. The name was derived from the conserved WD dipeptide and length of ∼40 amino acids in a single repeat. Generally, each WD40 repeat comprises a four-stranded antiparallel β-sheet, forming a strong hydrogen bond network to stabilize the WD40 repeat fold. The repeats fold into a β-propeller architecture in which the last three strands of the last blade form a β-sheet with the outer strand from the first blade, linking the beginning and end and creating a ‘velcro’ closure to further stabilize the propeller structure. This domain is involved in several functions, such as signal transduction, vesicular trafficking, cytoskeletal assembly, cell cycle control, apoptosis, chromatin dynamics, and transcription regulation. In most cases, it serves as a rigid scaffold for protein–protein interactions to co-ordinate downstream signaling events [17].

In some cases, the WD40 domain is flanked by a helical region at either the N-terminus or the C-terminus, as seen in Figure 1C. For example, one of the best-studied G proteins is the Rod transducin, GT_αβγ_. Upon activation, G_α_ dissociates from the G_βγ_ to perform further downstream signaling. The G_βγ_ are never seen dissociated from each other and function as a single entity (Supplementary Figure S1). The N-terminus of Gt_β_ is shown to begin with a helical structure followed by the canonical WD40 repeats (PDB ID: 1TBG) (Figure 1C, left panel) [18]. The exact function of this helix is not well understood, but it is implicated in binding effector molecules. Other WD40 domains have a C-terminal helix (coiled-coil), which in axonemal outer-arm dynein helps to attach it to microtubule doublets (PDB ID:7K58) (Figure 1C, right panel) [19]. Thus, the significance of the structure-function relationship for WD40-containing proteins is not well appreciated to date.

### WD40 domain of LRRK2: exploring the dynamics using HDX-MS and GaMD simulations

**Figure 2.**
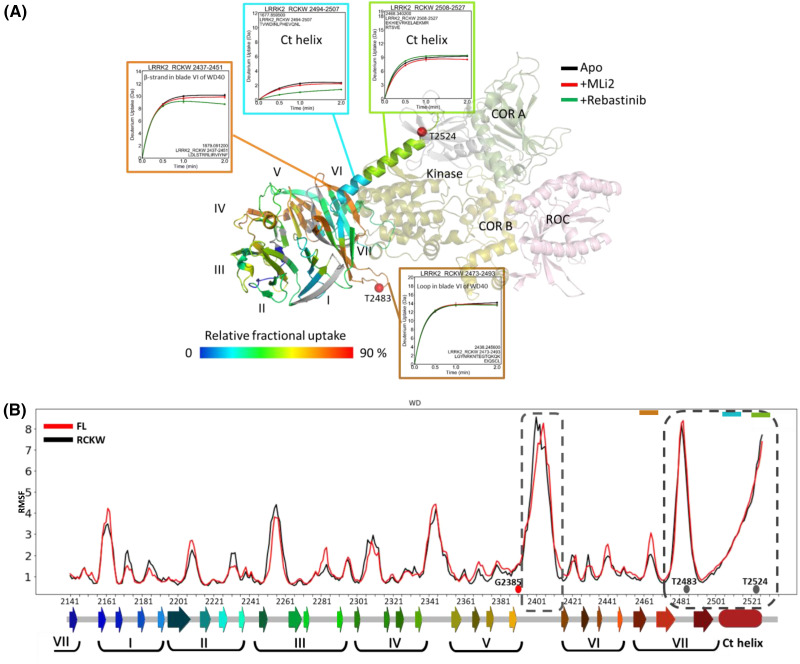
The deuterium uptake and Gaussian MD simulations of LRRK2 WD40 domain. (**A**) The WD40 domain of LRRK2 is mapped according to the hydrogen-deuterium exchange mass spectrometry (HDX-MS). The deuterium uptakes of the selected peptides are plotted focusing on the Ct helix. The presence of MLi-2 does not show a significant effect on uptake. In the presence of Rebastinib, N-terminus of Ct helix shows enhanced shielding from the solvent. (**B**) The RMSF analysis of WD40 domain generated from the GaMD simulations of full-length (FL, 7LHW) and RCKW (6VNO) LRRK2 shows that the loops between the blades V and VI are the most flexible regions along with the long loop in blade VII and the Ct helix. The colors at the bottom represent the structure of WD40 from N-terminus to C-terminus (blue through red).

The WD40 domain of LRRK2 has the canonical seven-blade architecture with the C-terminal helix extending from its C-terminus (Figure 1B,C, middle panel). The I-helix of the kinase domain is immediately followed by the first blade of the WD40, anchoring it to the kinase domain. This is seen in all the available cryo-EM structures of LRRK2. The function of the WD40 domain is not well understood but has been implicated in microtubule and synaptic vesicle interaction and neurotoxicity [20]. To understand the WD40 and kinase domain dynamics, we carried out HDX-MS and GaMD simulations of the RCKW (6VNO) protein. HDX-MS data shows the loop harboring the T2483 phosphorylation site is very flexible and solvent-accessible (residues 2473–2493) along with the last blade (blade VI) of the WD40. The beginning of the Ct helix is shielded from solvent (residues 2494–2507), while the other end is more solvent-accessible (residues 2508–2527) (Figure 2A). The presence of a type-I inhibitor, MLi-2, does not make a significant difference in the deuterium uptake of these regions. However, the presence of type-II inhibitor Rebastinib shows changes in peptide 2494–2507, making it more solvent-shielded. The overall uptake of the WD40 domain shows it to be a very stable structure, with only the loops between blades being solvent-accessible.

Our Root Mean Square Fluctuation (RMSF) results based on the GaMD simulations of full-length LRRK2 (FL, 7LHW) and RCKW show the β-sheets to be very stable, whereas the loops are more dynamic (Figure 2B). The loop between blades V and VI is one of the most flexible regions of the WD40. This is particularly interesting as this loop begins from the β-sheet harboring the PD mutation G2385. Another highly flexible loop based on HDX-MS and RMSF was between the last two β strands of blade VII, bearing the autophosphorylation site T2483. The Ct helix is also very flexible in both structures. This analysis also correlates well with the HDX-MS results from Figure 2A, with the flexible parts being more solvent-accessible. The least dynamic region in the WD40 domain, both FL and RCKW, is blade V. This is in accordance with the crystal structure of this domain [5], where it is shown to be a significant part of the dimer interface. The HDX-MS and GaMD simulations provide valuable insights into the structural dynamics of the WD40 domain. The findings suggest that the WD40 domain forms a stable scaffold composed of blades with dynamic loops connecting them. These loops exhibit varying conformations, sometimes positioned between individual strands within a blade and at other times positioned between different blades of the WD40 domain.

### Interactions with the kinase domain: capturing the dynamics between the Ct-helix and the kinase domain

**Figure 3.**
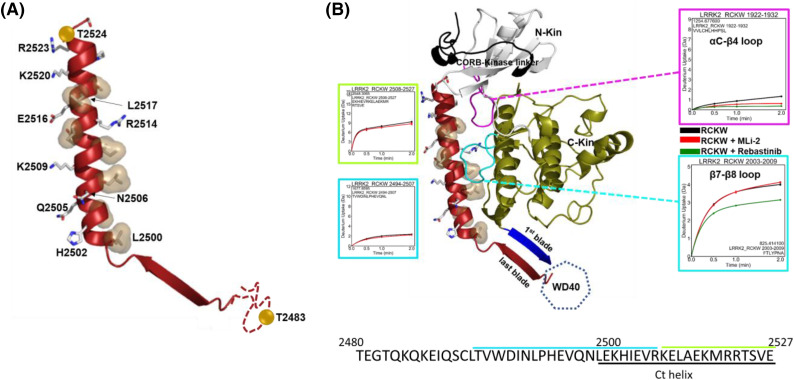
Interaction of the Ct helix with the kinase domain. (**A**) The highlighted residues along the Ct helix are involved in interaction with the kinase domain. It shows the amphipathic nature of the helix. The two phosphorylation sites are also marked as spheres in gold. (**B**) The Ct helix is stably docked onto the kinase domain with the first blade (blue) of WD40 extending from the I-helix in the C-lobe of the kinase. The deuterium uptakes of the αC-β4 loop in the N-lobe and β7-8 loop in the C-lobe are also shown. The β7-8 loop is more flexible but not effected by the presence of MLi-2 whereas the αC-β4 loop is shielded and becomes more so in the presence of MLi-2. In the presence of Rebastinib, the β7-8 loop becomes more protected. The deuterium uptakes of the Ct helix shown here again along with its sequence to emphasize that the Ct helix is well shielded from the solvent in the beginning as compared with its later half.

All the structures of LRRK2 available to date show the Ct helix to be docked onto its kinase domain spanning both the N- and C-lobes. As seen in Figure 3A, it is an amphipathic helix with its positively charged surface primarily interacting with the kinase domain. It makes both electrostatic and hydrophobic interactions with mainly the C-lobe of the kinase, some of which are shown in Figure 3B. Its negatively charged surface is solvent-exposed and can potentially interact with the NTDs or other effector molecules such as the Rabs.

Our previous HDX-MS study showed that the Ct helix and the kinase domain interface is well shielded from solvent based on low deuterium uptake [21]. The N-terminus of the Ct helix interacts with the α-I helix, the terminal helix in the C-lobe of the kinase core, and stably anchors the helix onto the kinase domain's C-lobe. At the same time, the C-terminus interacts with the N-lobe of the kinase domain as well as with the CORB-kinase linker. We also show the HDX-MS of the αC-β4 loop and the β7-8 loop of the kinase domain (Figure 3B). The β7-8 loop in LRRK2, which is longer than most kinases, is adjacent to the Ct helix. The deuterium uptake shows that the αC-β4 loop is very well shielded from the solvent, and the presence of MLi-2 and Rebastinib makes it even more shielded. The decrease in deuterium uptake upon MLi2 binding aligns with the recently reported MLi-2 bound LRRK2 structure [22,23], which shows that the binding of MLi-2 stabilizes the active conformation, making it more compact compared with the apo structure.

The β7-8 loop is more solvent-accessible when compared with the αC-β4 loop. The presence of Rebastinib makes it more shielded, while MLi-2 shows no significant change. The presence of Rebastinib also makes the N-terminus of the Ct helix more shielded from solvent. In a previous study, we demonstrated that the binding of Rebastinib stabilizes an open conformation of the LRRK2 kinase domain and promotes an extended structure of RCKW, resulting in the ROC: CORA domain being positioned further away from the kinase domain as compared with MLi-2 [24]. We hypothesize that the kinase domain interacts more strongly with the Ct helix in this open conformation. Simultaneously, the interactions between the kinase domain and the ROC:CORA domain are weakened.

The conformation of the αC-β4 loop is very stable and conserved through different kinases, including LRRK2. The hydrophobic tip of the loop is packed against the α-E helix and anchors the C-lobe [25]. In the case of LRRK2, the Ct helix packs against the αC-β4 loop and interacts with the α-E helix residue D1980 via R2514, while the loop residue H1929 bridges the interaction (Supplementary Figure S2). We hypothesize that the Ct helix can play an important role in the regulation of the kinase domain. It can be inhibitory when docked onto the kinase domain in FL LRRK2. The Ct helix can be unleashed by the binding of NTDs to Rab proteins, rendering the kinase domain active.

### LRRK2 C-terminal helix (Ct helix): peptide arrays and GaMD simulations

The Ct helix of LRRK2, which directly follows the WD40 domain, comprises residues 2500–2527. We predicted the helical propensity of this region before structure solutions (Supplementary Figure S3A), which was confirmed by all the LRRK2 structures known to date. The function of this Ct helix of LRRK2 is unknown. Still, this region, in addition to being anchored firmly to the kinase domain, has a few critical phosphorylation sites and localization motifs. To gain a deeper understanding of its interactions with the rest of the LRRK2 domains, we recombinantly expressed residues 2480–2527 (Supplementary Figure S4), which includes the last WD40 blade and the C-terminal helix of LRRK2. This region contains two autophosphorylation sites: T2483 and T2524 [26]. T2524 is known to be a putative 14-3-3 binding site [27].

**Figure 4.**
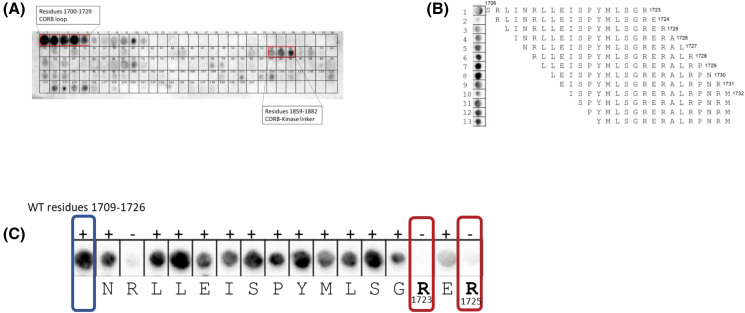
Peptide array analysis of Ct helix binding to the CORB linker. (**A**) The CORB and kinase domains of LRRK2 (residues 1700–2140) were arrayed as 18-mers that were staggered by three residues. Overlay of these peptides with the Ct-helix motif identified several interacting residues but most prominent were a set of peptides at the beginning of the CORB domain (residues 1709–1732). Peptides corresponding to this region were then arrayed and staggered by a single residue in panel (**B**). (**C**) An Alanine scan was then carried out for one of these peptides where each residue was replaced by Ala. Replacement of R1723 and R1725 with Ala abolished binding indicating that these were two essential parts of the binding motif.

We performed an overlay analysis to investigate the interactions between LRRK2 domains and the purified Ct helix protein. Specifically, we overlaid the CORB and kinase domains (residues 1700–2140) with the purified Ct helix protein. This analysis revealed that the loop region spanning residues 1700–1727 in the CORB domain (now referred to as the CORB loop) interacts with the Ct helix peptide (Figure 4A,B). The Ct helix also interacts with the CORB-kinase linker region, residues 1862–1883 (Figure 4A). This region was consistently identified in a subsequent array where we used regions 1706–1730 on the array and overlayed with the Ct helix protein. We then performed an Alanine scan of the most reproducible region from 1709 to 1726 and identified R1723 and R1725 as potential important interaction sites with the Ct helix. Mutating either arginine to Ala abolished interaction with the Ct helix (Figure 4C). When R1723 and R1725 are converted to an Ala simultaneously, no interaction with the Ct helix was observed (Data not shown). This led us to use GaMD simulations to explore this region. We carefully dissected the domain dynamics in FL and RCKW structures.

**Figure 5.**
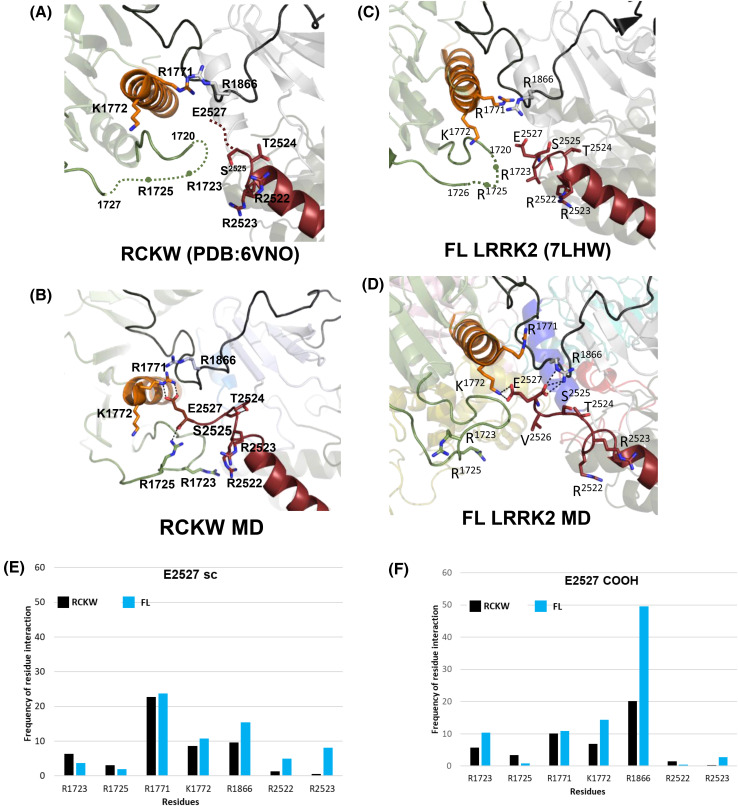
Interactions of E2527 in LRRK2 RCKW and FL revealed by GaMD simulations. (**A**) The cryo-EM structure of LRRK2 RCKW (PDB:6VNO). Residues 1721–1726 and 2526–2527 are undiscernible and were missed in the model. (**B**) Snapshot from the GaMD simulations of LRRK2 RCKW with modeled missing residues showing the terminal residue E2527 is interacting with the R1725 and R1771 on the CORB. (**C**) The cryo-EM structure of FL LRRK2 (PDB:7LHW). Residues 1721-1725 are undiscernible and were missed in the model. (**D**) Snapshot from the GaMD simulations of FL LRRK2 showing the E2527 is interacting with K1772 and on the CORB and R1866 on the CORB-kinase linker. The bar graphs display the average frequency of residue interactions with the side chain (**E**) of E2727 and carboxyl groups (**F**) of E2527 over three repetitions of 200 ns simulation time.

In the RCKW (6VNO) and FL (7LHW) structures of LRRK2, the CORB loop region (1700–1727) is unstructured (Figure 5A,C). The C-terminus of the Ct helix lies in close proximity to the Dk helix in CORB (orange helix in Figure 5), the CORB loop (shown in green), and the CORB-kinase linker (shown in black) in both RCKW and FL LRRK2. The last three residues of the Ct helix in the RCKW cryoEM structure are missing while forming the helix's last turn in the available FL structures. To conduct the GaMD simulations, we modeled the missing residues into the RCKW cryo-EM structure.

The simulations show the carboxy terminus of the Ct helix is positioned between the CORB domain and the N-lobe of the kinase domain. Based on our simulations, the two arginine residues, R1723 and R1725, in the long loop of the CORB domain form H-bonds with E2527 (Figure 5B). Our peptide array studies also identified these arginines as critical for the interaction between the CORB and the Ct helix. Another arginine, R1866, on the CORB-kinase linker, can also interact with E2527 (Figure 5D) in the RCKW and FL LRRK2. As reported previously, R1771 and K1772 at the end of the CORB helix could stabilize the Ct helix terminal residues (2522–2527) [21]. The frequency of interactions of these residues with the sidechain and α-COOH of E2527 is shown in Figure 5E and F, respectively. The E2527 sidechain appears to be quite flexible; it interacts weakly with several residues including R1771 and R1866 in both RCKW and FL LRRK2. In contrast, in FL LRRK2, and not in RCKW, the carboxy group of E2527 primarily interacts with R1866. In particular, Figure 5E illustrates the sidechain of E2527 engaging weakly with R1771, located on the CORB loop, and R1866, on the CORB-kinase linker, but there is no difference between FL LRRK2 and RCKW. There is a slight increase in the interaction frequency with R1866 in the FL form compared with RCKW as well as with R2522 and R2523, but overall, the simulations suggest no dominant interaction. In contrast, Figure 5F highlights strong interactions of E2527's carboxyl group with R1866, as well as a weaker preference for R1722, and these interactions, which link the C-terminus to the CORB-kinase linker and the CORB domain, are unique to FL LRRK2. These interactions suggesting a more pronounced association of the C-terminal helix with the CORB and kinase domains in the FL LRRK2 may explain why deletion of only a few residues at the C-terminus destabilizes LRRK2.

These interactions provide insight into how the C-terminal tail can potentially mediate both positive and negative cross-talk between the kinase and the GTPase domains. While the Ct helix interacts with the N-terminus of the CORB helix, the C-terminus of this helix lies at the interface between CORB and the ROC GTPase domain. There is a dynamic interplay of the terminal residues of the Ct helix communicating with the N-terminus basic residues of the CORB helix.

### CORB domain dynamics

The cross-talk between the Kinase and CORB domains of LRRK2 has been previously carefully dissected. We have defined how the CORB helix (Dk helix, residues 1771–1791) co-ordinates communication between the ROC and the kinase domain [21]. The kinase domain is tethered to both the WD40 domain and the Ct-helix. The catalytically inert COR domain consists of two subdomains, CORA and CORB, connected by a flexible linker. The CORA domain is firmly linked to the ROC domain, while the CORB domain interacts with the kinase domain, the ROC domain, and the Ct helix. The CORB helix is positioned against the αC helix in the N-lobe of the kinase domain, and its C-terminal segment approaches the disordered activation loop of the kinase domain and the ROC domain. Therefore, the CORB domain facilitates allosteric communication between the ROC and the kinase domain.

**Figure 6.**
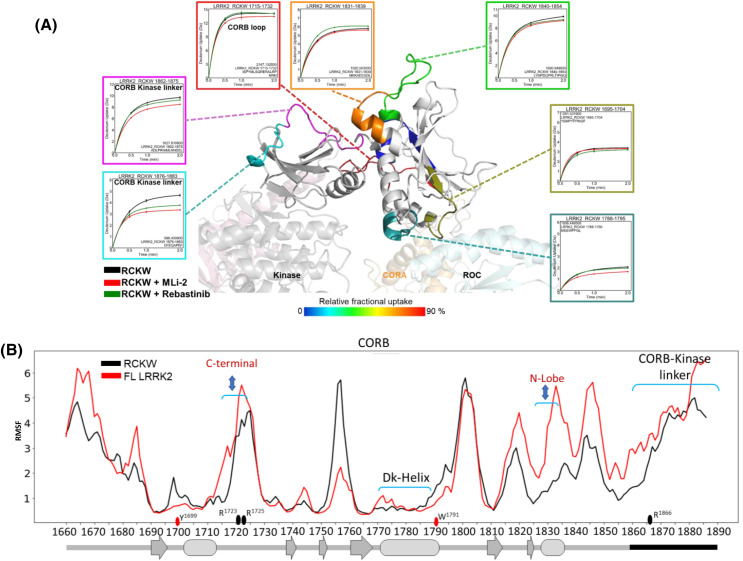
The deuterium uptake and Gaussian MD Simulations of LRRK2 CORB domain. (**A**) The CORB domain of LRRK2 is mapped according to the HDX-MS. The deuterium uptakes of the selected peptides are plotted on the CORB domain in the presence of MLi-2 and Rebastinib. (**B**) The RMSF analysis of CORB domain generated from the GaMD simulations of full-length (FL, 7LHW) and RCKW (6VNO) LRRK2 shows that the FL is more flexible in the loop region that interacts with the Ct helix and the CORB-kinase linker.

Here, we focus on two regions: the CORB loop (residues 1700–1727) and the CORB-kinase linker (residues 1862–1883). These regions were identified through peptide array interactions and GaMD simulations. The deuterium uptake analysis reveals that both regions are accessible to solvent (Figure 6A). Interestingly, the presence of inhibitors does not significantly impact the deuterium uptake in the CORB loop. However, the CORB-kinase linker shows increased shielding in the presence of inhibitors, indicating a change in its solvent accessibility (Figure 6A).

The RMSF analysis of the CORB region in FL and RCKW LRRK2 reveals an order-disorder transition in the CORB-kinase linker; specifically, the CORB-kinase linker exhibits less flexibility (more ordered) in the RCKW structure compared with FL LRRK2. In FL LRRK2, the LRR domain wraps around the kinase domain, sterically shielding the active site, thereby rendering LRRK2 inaccessible to substrate binding [8]. The RCKW structure is closer to an active FL LRRK2, where its NTDs are unleashed. Analyzing the dynamics of RCKW and FL LRRK2 provides valuable insights into the mechanism of LRRK2 activation. Additionally, the CORB loop exhibits greater flexibility in the FL structure than RCKW. Understanding these differences in dynamics sheds light on the functional implications and regulatory mechanisms of LRRK2.

### Phosphorylation induced dynamics in LRRK2

We performed GaMD simulations of RCKW in its apo-form and when it is phosphorylated at T2483 (pT2483) or T2524 (pT2524) in the Ct helix. We compared the RMSF analysis of these simulations in different domains of LRRK2 and how it affects the interactions with the Ct helix (Supplementary Figure S6). Firstly, we observed differences in the helical stability of the Ct helix. In the FL LRRK2, the Ct helix remains stable throughout the simulation. However, we observed fluctuations in the last five residues throughout the RCKW simulations. In the case of pT2524 simulations, these residues lost their helical structure and did not maintain a helix (Supplementary Figure S3B).

When the T2524 is phosphorylated in the RCKW simulations, the E2527 sidechain interacts with R1771, similar to the apo form. The -COOH of E2527 makes most interactions with R1771 and R1866 (Supplementary Figure S5A,C,D). The sidechain of T2524 mainly interacts with R2522 and R2523 when phosphorylated (Supplementary Figure S5E). For the phosphorylation at T2483, the E2527 sidechain mainly interacts with R1771, similar to the apo-form and pT2524, while the –COOH of E2527 still interacts with R1771 in addition to R1866 (Supplementary Figure S5B,C,D). Interestingly, phosphorylation at T2524 makes the C-terminal helix more dynamic than the apo RCKW, opening potential interactions for 14-3-3 proteins.

For the WD40 domain, the RMSF analysis shows the loop between βI and βII of strand III is more dynamic in the apo-form than in the phosphorylation states, while the loop between strands V and VI is more stable in the apo-form. The end of the WD40 and the beginning of the Ct helix (residues 2500–2521) become more dynamic in the phosphorylation states than in the apo-form, the pT2524 being the most dynamic (Supplementary Figure S6A). These results indicate that the strategically placed phosphorylation and 14-3-3 binding sites (T2483 and T2524) at the Ct helix motif may mediate interactions with the NTDs in full-length LRRK2.

Moving on to the kinase domain (Supplementary Figure S6B), phosphorylation at T2483 and T2524 induces greater flexibility in the C-lobe (particularly in the region from αG to αI). This effect is even more pronounced when the Ct helix is phosphorylated at T2524. In the N-lobe, we see more flexibility in the case of pT2524 in residues 1865–1880, which form a part of the CORB-kinase linker.

We also compared the ROC:CORA and CORB dynamics in the RCKW structure, both in the apo form and when phosphorylated (as shown in Supplementary Figure 6C,D). In the ROC:CORA region, phosphorylation increases flexibility compared with the apo-form. This effect is particularly pronounced in the Switch I region and the regions interacting with the LRR-ROC linker (Supplementary Figure S6D). In the CORB, we see increased flexibility in the CORB loop region that interacts with the Ct helix. Based on our RMSF analysis, the altered interactions and dynamic behavior suggest that phosphorylation at T2483 modulates the conformational state of the kinase, which in turn could affect its enzymatic activity and interaction with other domains, particularly the CORB domain. This phosphorylation event likely plays a crucial role in regulating LRRK2's kinase activity and its role in cellular processes.

Overall, we see increased flexibility in most domains when the Ct helix is phosphorylated at T2524, indicating this site may be an important regulation site for the overall dynamics and, consequently, the function of LRRK2.

## Discussion

The WD40 domain of LRRK2 extends into a stable Ct helix, anchored to the kinase domain in all available structures to date. This helix has a strong helical propensity. The Ct helix is rich in small linear motifs (SLiMs) and phosphorylation sites, rendering it a hotspot for multiple intra- and intermolecular protein–protein interactions.

The Ct helix can be divided into an N-terminal region, which is always stably anchored to the WD40 domain, and the C-lobe of the kinase domain, very shielded from the solvent. In comparison, the C-terminal end of the helix is intrinsically disordered, positioned close to the NTDs, especially the hinge helix, and is shown to interact with the ARM domain through cross-linking studies [9,13]. The Ct helix region is also crucial for the kinase activity of LRRK2 [11,12]. The C-terminal end of the Ct helix is also rich in negative charges: a terminal negatively charged amino acid E2527, the carboxy terminus of LRRK2 itself, and the potential to be phosphorylated at T2524 and/or S2525. We also point out that S2525 can be a potential PKA phosphorylation site as it has the canonical consensus motif (RXXS/T: R^2522^XXS^2525^). PKA phosphorylation sites in LRRK2 have been mapped using mass spectrometry and effect of these phosphorylation has been studied in terms of 14-3-3 binding [28].

**Figure 7.**
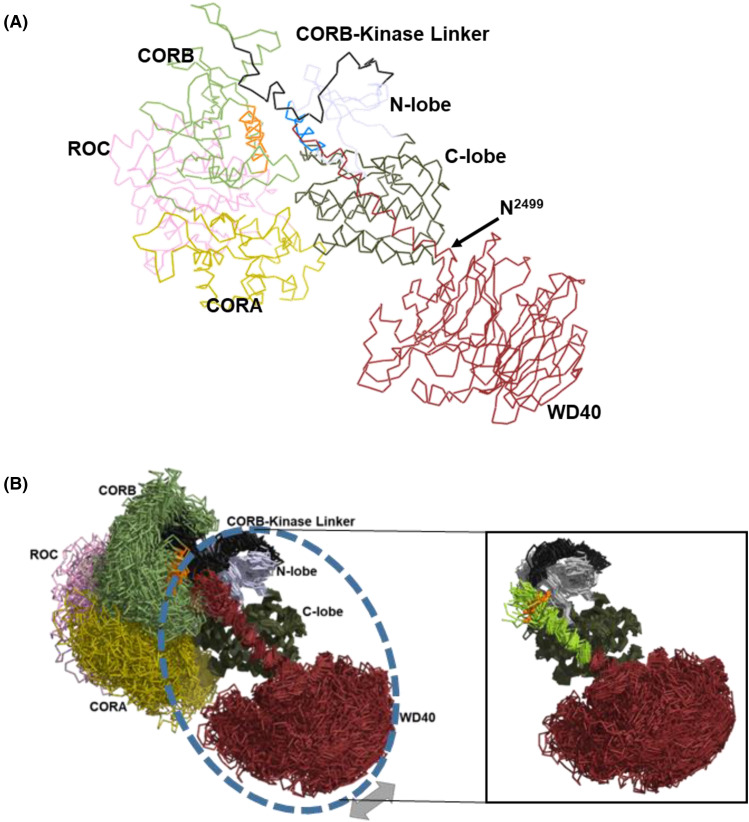
Domain Movements of LRRK2 RCKW revealed by GaMD. (**A**) Ribbon representation of LRRK2 RCKW highlighting the hinge between the C-lobe of the kinase and WD40. The Ct-helix spans across the kinase domain, and the C-terminal is in close proximity to CORB and CORB-kinase linker. (**B**) An ensemble of structures from GaMD simulations aligned by the α-E and α-F helices of the kinase domain over a simulation time of 200 ns. The simulation shows that the WD40 moves as a rigid body relative to the C-lobe of the kinase domain, while the Ct helix is anchored to the C-lobe and move as the same group as the C-lobe. The CORA, CORB, and ROC domains all move freely relative to the C-lobe of the kinase. The inset shows the C-terminal half of the Ct helix is more flexible in the simulations, it is colored according to the deuterium uptake of the peptide with the T2524 marked as a orange sphere.

Our GaMD simulation and peptide array results show that the C-terminal end of this helix interacts with a CORB loop (1720–1727) that is disordered in all known cryoEM structures. R1723 and R1725 are shown to be critical residues mediating cross-talk between this loop and the Ct helix. Additionally, this CORB loop region is in proximity to the N-terminus of the CORB helix. This pivotal motif anchors the CORB domain to the αC helix of the kinase domain [21]. Together, the Ct helix, the CORB loop, and the CORB helix form a ‘CAP’ for the N-lobe of the kinase domain, mediating cross-talk between the GTPase and kinase of LRRK2. This group also includes the CORB linker to the kinase domain (Figure 7A). This CORB-kinase linker undergoes an order-disorder transition when comparing RMSF analysis of FL and RCKW LRRK2. It is more flexible in the FL, where the NTDs are present, which might indicate a loose control of cross-talk between the CTDs. In contrast, it becomes more ordered in RCKW to mediate communication between the GTPase and the kinase domains. R1866 from this CORB-kinase linker interacts with E2527 of the Ct helix in simulations of FL LRRK2. In both FL and RCKW LRRK2 (Figure 4A–D), we see a concentration of positive charges (R1723, 1725, 1771, and 1866) from different areas of LRRK2 in close proximity to the negatively charged end of Ct helix (E2527, pT2524, potentially pS2525, and COOH–). These electrostatic interactions under different conditions could be critical in mediating cross-talk between the kinase and GTPase domains of LRRK2 and most likely explain why deleting a few residues at the C-terminus renders the kinase inactive [11,12].

Utilizing the structural information from FL [8,9] along with GaMD, we categorize five rigid bodies in LRRK2: the C-lobe of the kinase domain with the stably anchored Ct helix; the β-propeller structure of WD40 which moves independently relative to the C-lobe of the kinase domain; the N-lobe of the kinase domain; CORB domain, which functions as a separate rigid body interacting with the kinase domain, ROC and CORA domain; and the ROC: CORA domain moving as a unit (Figure 7B). These rigid bodies move in a co-ordinated manner, regulating the transition of LRRK2 from an inactive, extended conformation (where the ROC domain is far from the kinase domain) to an active conformation with close proximity between the ROC and C-lobe of the kinase. These conformation changes demonstrate the cross-talk between the kinase and GTPase domains.

The most recent structure of active FL LRRK2 [29] also describes a complex tetrameric state of the protein. This state exhibits a two-fold symmetry consisting of two central, active, and two peripheral, inactive LRRK2 protomers. The central LRRK2 protomers pack against each other in a ‘head to tail’ fashion, bringing the Ct helices from the two protomers antiparallel to each other (Supplementary Figure S7). This opens door to a new set of potential interactions in this region, precisely when the Ct helix may be phosphorylated at T2524/S2525 and/or bound to 14-3-3 proteins [27]. This region could potentially serve as a dimer interface and become one of the several factors managing the active/inactive conformation of the kinase domain. Our simulations conducted on the RCKW, and its variants show that the last five residues do not sample as a helix in the pT2524 state (inset, Supplementary Figure S3), rendering this region more dynamic. The distance between the two antiparallel Ct helices in this region is ∼38 Å. We hypothesize that this can be a potential 14-3-3 dimer binding region. Because the Ct helix can be auto phosphorylated, between the two active protomers we speculate that the potential dimer interface allows 14-3-3 to regulate LRRK2 function. It could be another allosteric region for LRRK2 regulation.

Additionally, this region includes two ER-localization motifs (SLiMs): a di-lysine(K^2485^QK^2487^) and a di-arginine (MR^2522^R^2523^) motif. A signal sequence generally accompanies the targeting of proteins in the ER. The di-lysine motifs are usually present at the carboxy-terminal of cytoplasmic proteins and are sufficient for retaining such proteins in the ER of mammalian cells. There is a requirement for two Lys residues at the carboxy-terminal. Mutating these Lys residues to Ser destroys the targeting motif [30]. More recently, the role of such a di-Lys motif has been implicated in the retrograde trafficking of the SARS-CoV virus [31].

Similarly, a di-Arg motif was also identified in some ER-resident type II membrane proteins [32]. These motifs are also present in the C-terminal helix of LRRK2. These motifs are generally regulated by stearic masking: binding of another subunit partner or by binding 14-3-3 proteins (T2524 is a 14-3-3 binding site) and the presence of potential phosphorylation sites (S2525) in its vicinity [26,27]. The LRRK2 Ct helix motif fulfills all these criteria of being localized to the ER and/or negatively regulated by phosphorylation sites. Experimental evidence of whether the Ct motif is sufficient for ER localization is beyond the scope of this manuscript and may be followed up in another study.

The Ct helix can also be compared with the A-helix of PKA C-subunit, an amphipathic helix that docks onto the kinase core. The hydrophilic surface of the A-helix binds to A kinase-interacting protein [33]. This scaffolding protein, in turn, can bind to both the catalytic and regulatory subunits of PKA and play an essential role in the regulation and localization of PKA [34]. Previous studies have focused on the vital role of the Ct helix in regulating LRRK2's kinase activity; in this study, we explore the interactions of the Ct helix and its function in bridging the CORB and kinase domains. This capping motif for the N-lobe of the kinase domain is a critical site for regulation in many kinases such as BRAF which is a close homolog of LRRK2. The Ct helix clearly has the potential to significantly impact the intramolecular network that governs the kinase and ROC domains, and phosphorylation of the Ct helix can distinctly alter LRRK2's networks and dynamics, directly influencing LRRK2's kinase or GTPase activity. These findings underscore the pivotal role of the C-terminal tail in LRRK2's regulatory mechanisms and in mediating intra-domain cross-talk.

## Methods

### Protein purification

The Ct helix (residues 2480–2526) was cloned into the pET-His6 LIC cloning vector (Addgene #29659). The construct was transformed into BL21(*DE3*) cells for expression and induced by 1 mM IPTG when O.D._600_ reached 0.6. After 16-h expression at 37°C, the bacterial pellets were collected. The pellets were re-suspended and lysed in the lysis buffer: 20 mM Tris–Cl, 300 mM NaCl, 2 mM DTT. The supernatant was collected after high-speed centrifugation (13 000 rpm, 1 h) and passed through the Ni-resin. The resin was then washed with three column volumes of wash buffer (20 mM Tris–Cl pH = 8.0, 300 mM NaCl, 10 mM imidazole, 2 mM DTT), and the Ct helix was eluted by elution buffer (20 mM Tris–Cl pH = 8.0, 300 mM NaCl, 500 mM imidazole, 2 mM DTT). This protein was further purified by S-75 gel filtration column with buffer 20 mM Tris pH = 8, 300 mM NaCl, 2 mM DTT.

### Peptide array

Peptide spot arrays were generated by the INTAVIS AG peptide synthesizer (INTAVIS Bioanalytical Instruments AG, Koeln, Germany) using standard Fmoc (9 fluorenylmethoxycarbonyl) protection-based solid phase peptide synthesis to produce short, overlapping 18-mer peptides directly conjugated onto amino-PEG modified cellulose membranes (ACS01, INTAVIS AG). Alanine scans were similarly generated by replacing each residue with Ala one at a time. After activation (incubation with ethanol for 5 min), the array was blocked with 5% BSA in TBS-T (TBS, pH 8.0/0.05% Tween 20) for 2 h at room temperature. The array was then washed three times with TBS-T for 10 min, followed by incubation overnight at 4°C with the purified Ct helix (His-tagged). This was followed by washing three times for 10 min with T-TBS at room temperature and incubation with anti-His antibody (1:500). After another round of washing, the array was incubated with a second anti-mouse IgG HRP conjugate secondary antibody (Genesee Scientific catalog no. 20-303) (1:5000) for 45 min at room temperature. This was followed by washing three times for 10 min with T-TBS. The final analysis used a chemiluminescence substrate and the ChemiDoc MP imaging system Bio-Rad.

### GaMD simulation

The simulation models for RCKW and fl-LRRK2 were prepared using cryo-EM structures (PDB: 6VNO and 7LHW). The missing loops in the protein structure were modeled using Modeller [35], the full system was parameterized using LEaP in AMBER16. Hydrogens and counter ions were added, and the resulting models were solvated in a cubic box of TIP4P-EW water molecules and 150 mM KCl with a 10 Å buffer using AMBER tools [36]. The systems were minimized through various steps, including hydrogen-only minimization, solvent minimization, ligand minimization, side-chain minimization, and all-atom minimization. The heating process involved two stages: first from 0 to 100 K under constant volume conditions, over 50 ps, using 2 fs time-steps and 5.0 kcal/mol/Å position restraints. Then, the temperature was increased from 100 to 300 K under constant pressure conditions, over 200 ps, while maintaining 5.0 kcal/mol/Å position restraints on the protein, which were controlled using the Langevin thermostat. For equilibration, a constant pressure simulation was conducted with a 10 Å non-bonded cut-off, during which protein restraints were applied for 500 ps. This was followed by an additional 300 ps of unrestrained equilibration. To enhance conformational sampling and explore different states, GaMD was utilized with GPU-enabled AMBER16 [37,38]. GaMD applies a Gaussian-distributed boost energy to the potential energy surface, accelerating transitions between meta-stable states while allowing accurate reweighting. Both dihedral and total potential boosts were used simultaneously. GaMD simulations involved collecting potential statistics for 2 ns, followed by 2 ns of GaMD during which boost parameters were updated. Each GaMD simulation was further equilibrated for 10 ns. Finally, for each construct, a minimum of three independent replicates, each running for 200 ns, were performed for GaMD, providing the conformational exploration results.

### Hydrogen-deuterium exchange mass spectrometry

RCKW proteins were expressed and purified from Sf9 cells [39]. Measurements were performed using a Waters Synapt G2Si system with nanoACQUITY UPLC and H/DX technology. The RCKW concentration was 5 µM in LRRK2 buffer (pH 7.4) containing 20 mM HEPES/NaOH, 800 mM NaCl, 0.5 mM TCEP, 5% Glycerol, 2.5 mM MgCl_2_, and 20 µM GDP. Deuterium uptake was measured in the presence and absence of kinase inhibitors MLi-2 (50 µM) or Rebastinib (50 µM). Peptide identification and analysis were performed using PLGS 3.0 and DynamX 3.0 (Waters Corporation) as described in previous publication. The HDX-MS data included at least three technical replicates. Data were corrected for back-exchange using a global back exchange correction factor. Deuterium uptake plots were generated using DECA (github.com/komiveslab/DECA), with data fitted using an exponential curve for visualization.

## Data Availability

The authors confirm that the data supporting the findings of this study are available within the article and its supplementary materials. Further requests can be made to the corresponding author.

## Open Access

Open access for this article was enabled by the participation of University of California in an all-inclusive *Read & Publish* agreement with Portland Press and the Biochemical Society under a transformative agreement with UC.

## Abbreviations

ANK: ankyrin
ARM: armadillo
CTD: C-terminal catalytic domain
GaMD: Gaussian Accelerated Molecular Dynamics
HDX-MS: Hydrogen-Deuterium eXchange Mass Spectrometry
LRRK2: leucine-rich repeat protein kinase 2
NTD: N-terminal interaction domain
PD: Parkinson's disease
RMSF: Root Mean Square Fluctuation
ROC: Ras-of-complex
SLiM: small linear motif
