# Supplementary material for: Role of the leucine-rich repeat protein kinase 2 C-terminal tail in domain cross-talk
Source: Biochem J. 2024 Feb 19;481(4):313–27. doi: 10.1042/BCJ20230477 (PMC10903466; doi:10.1042/BCJ20230477)
Supplement: Supplementary Material [file BCJ-481-313-s1.pdf]

Supplementary Figures with legends

Role of the LRRK2 C-terminal tail in domain crosstalk

Pallavi Kaila Sharma[1], Jui-Hung Weng[1], Jascha T. Manschwetus[2], Jian Wu[1], Wen Ma[3], Friedrich W. Herberg[2], Susan S. Taylor[1,3*]

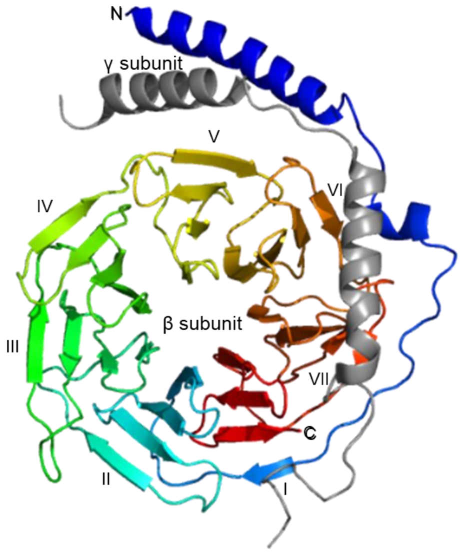

Supplementary figure 1 Domain organization of WD40 G-protein.

(A) WD40 domain with an extended N-terminal helix from a *Bos taurus* G protein (PDB ID: 1TBG) is shown in rainbow colors depicting the canonical seven-blade architecture from N- to C-terminus. This constitutes the β subunit of the G protein. In gray is the γ subunit of the G protein associated with the WD40 domain

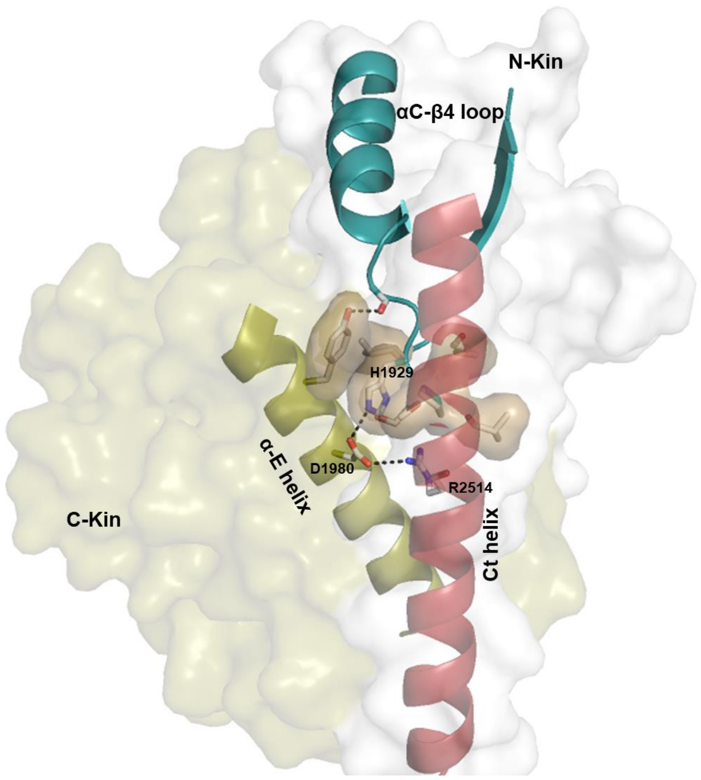

Supplementary figure 2 The conserved αC-β4 loop bridges the kinase core and the Ct helix
H1929 in the hydrophobic tip of the conserved αC-β4 loop bridges the interaction between the αE-helix (residue D1980) and the Ct helix (residue R2514).

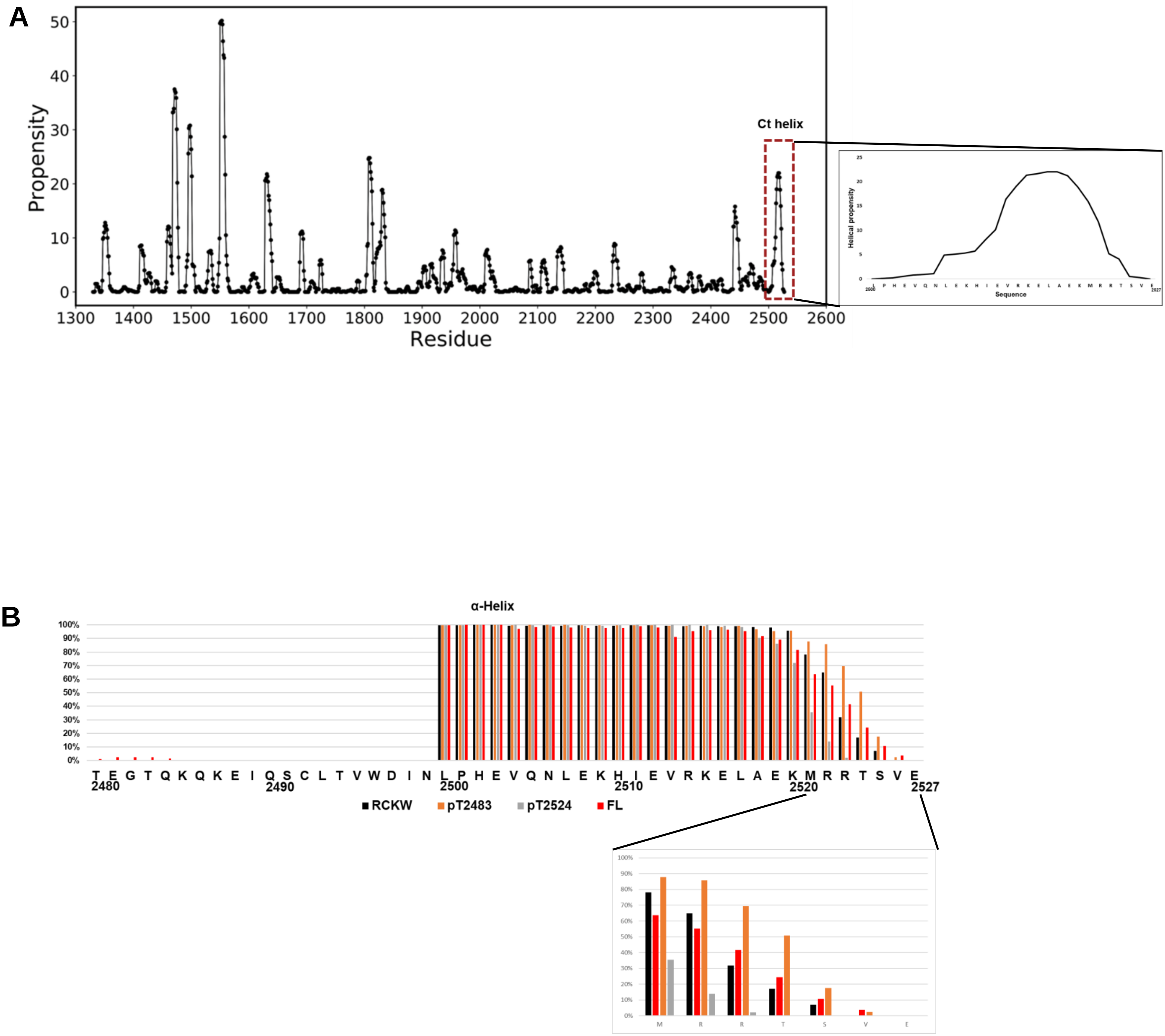

Supplementary figure 3 Helix propensity of the Ct helix.

(A) The helix propensity throughout the RCKW domains is shown. The helical propensity of the Ct helix is marked in the red box also shown in the inset.

(B) The helical propensity of the Ct helix motif based on GaMD simulations, including the last blade of WD40 is shown across the FL and RCKW construct and phosphorylation at T2483 or 2524. The propensity of last seven residues is shown in the inset.

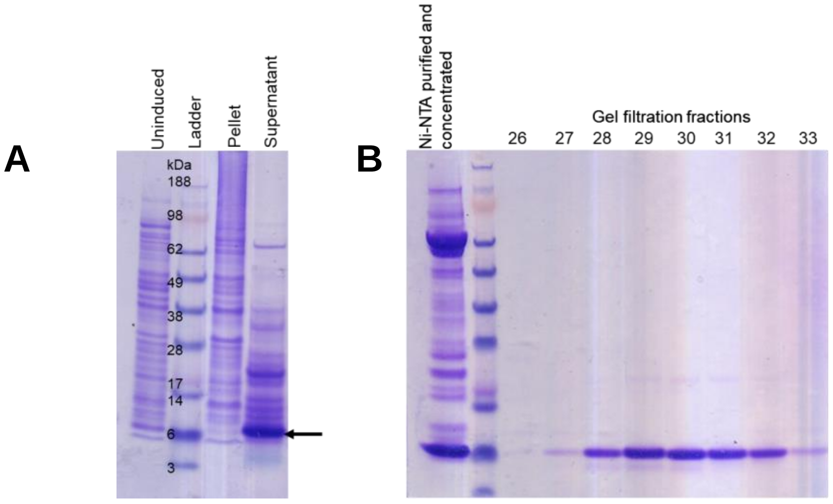

Supplementary figure 4 The purification of the Ct helix from *E.coli.*
(A) SDS PAGE shows the expression of the Ct helix in the supernatant fraction (shown by the arrow) of E. coli cell lysate. The expected size of the protein is ~7.1 kDa.
(B) SDS PAGE shows the Ni-NTA purified and concentrated sample of the Ct helix followed by the fractions obtained after passing it on a size-exclusion chromatography column.

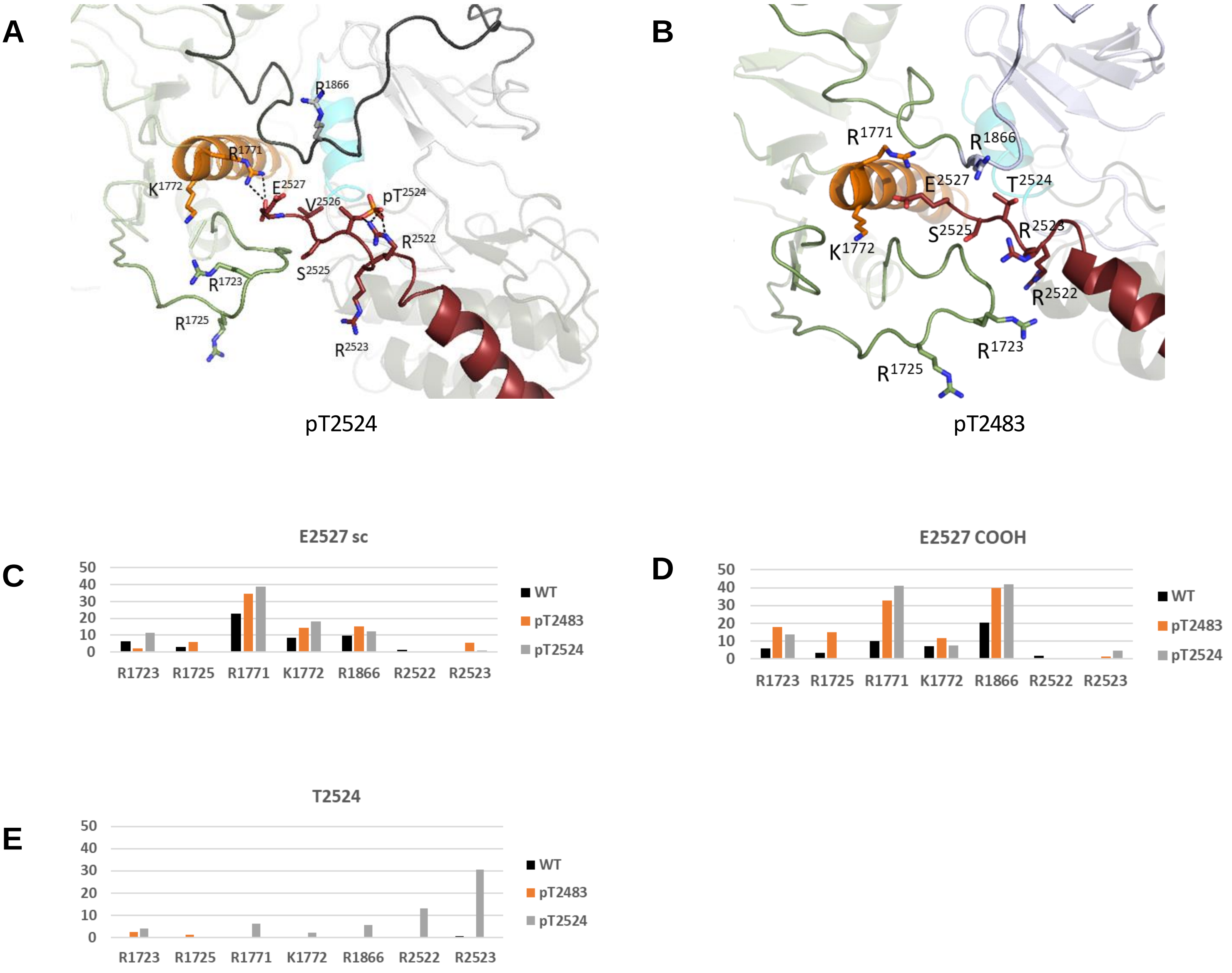

Supplementary figure 5 Interactions of E2527 in RCKW simulations with phosphorylation at T2524 or T2483.

(A) Snapshot from the GaMD simulations of LRRK2 RCKW with modeled missing residues and T2524 phosphorylated, showing the terminal residue E2527 is interacting with R1771 on the CORB.

(B) Snapshot from the GaMD simulations of LRRK2 RCKW with modeled missing residues and T2483 phosphorylated, showing the terminal residue E2527 is interacting with the R1725 and R1771 on the CORB.

(C) and **(D)** are bar graphs displaying the average frequency of residue interactions of the side chain of E2727 and its carboxyl group when either T2425 is phosphorylated or T2483 is phosphorylated over 3 repetitions of 200 ns simulation time.

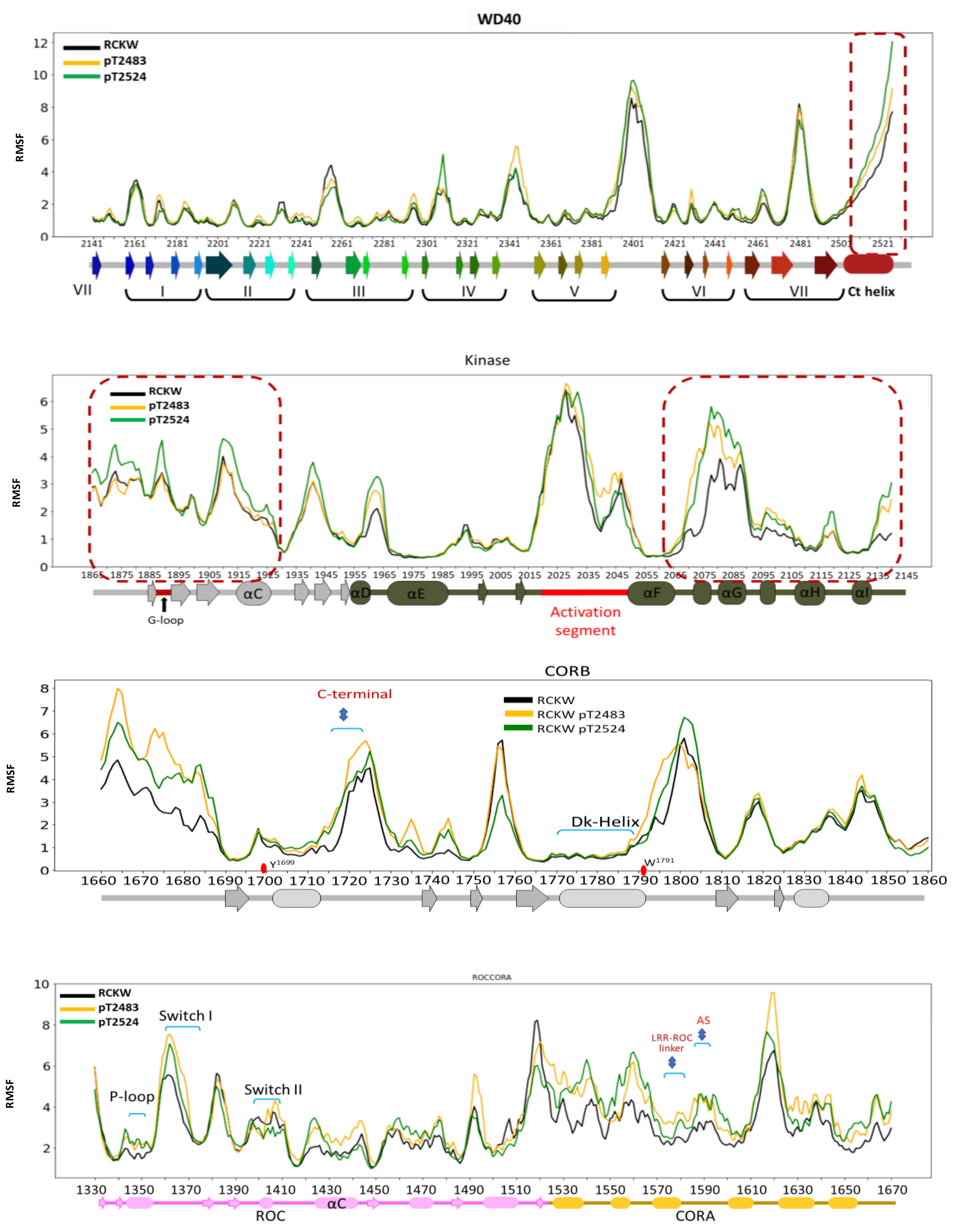

Supplementary figure 6 The RMSF analysis from Gaussian MD Simulations of LRRK2 RCKW and effect of phosphorylation at T2483 and T2524.

(A) The RMSF analysis of the WD40 domain in RCKW structure shows it to be a stable structure with the Ct helix increasing in flexibility by phosphorylation at T2524.

(B) The RMSF plots in the kinase domain show that phosphorylation at T2524 renders the RCKW structure more flexible in the G-loop region of the N-lobe and the αG/αH region of the C-lobe.

(C) The RMSF plots reveal that phosphorylation at T2483 and T2524 increases the dynamics of the loops at the N-terminus of the CORB domain but has less effect at the C-terminus of the CORB domain and the CORB to kinase linker.

(D) The RMSF analysis of the ROC:CORA domain shows that phosphorylation at T2483 and T2524 renders the overall ROC:CORA domain more flexible than unphosphorylated LRRK2, particularly in the P-loop and Switch I region of the ROC domain, and the CORA domain that is in close proximity with the activation segment and the region that potentially interacts with the LRR-ROC linker in the full-length construct.

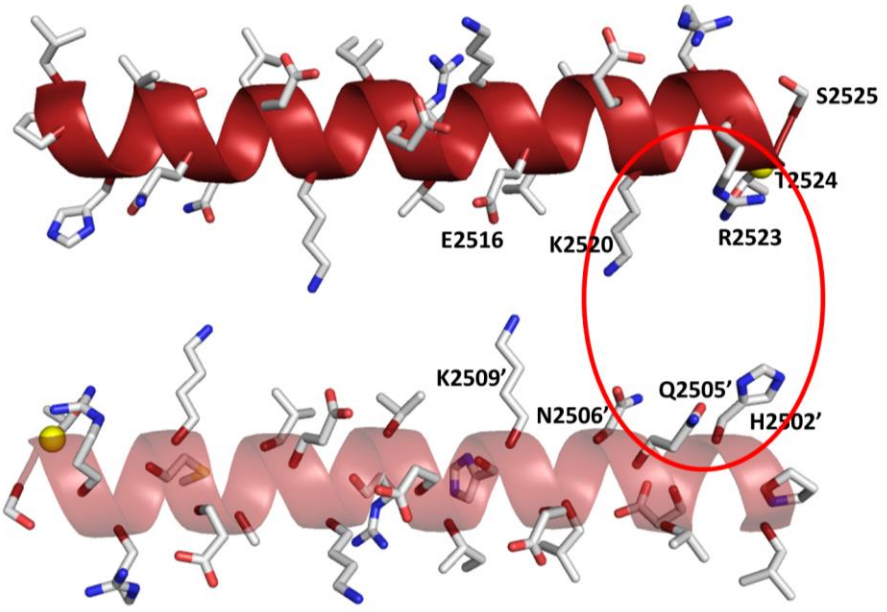

Supplementary figure 7 Antiparallel placement of the Ct helix
The most recent FL LRRK2 structure reveal the Ct helix are placed antiparallelly to each other due to the ‘head to tail’ alignment of LRRK2 dimer. This could be another potential dimer interface in LRRK2 because of the ability of the Ct helix to phosphorylate at T2524 and bind to 14-3-3 proteins.

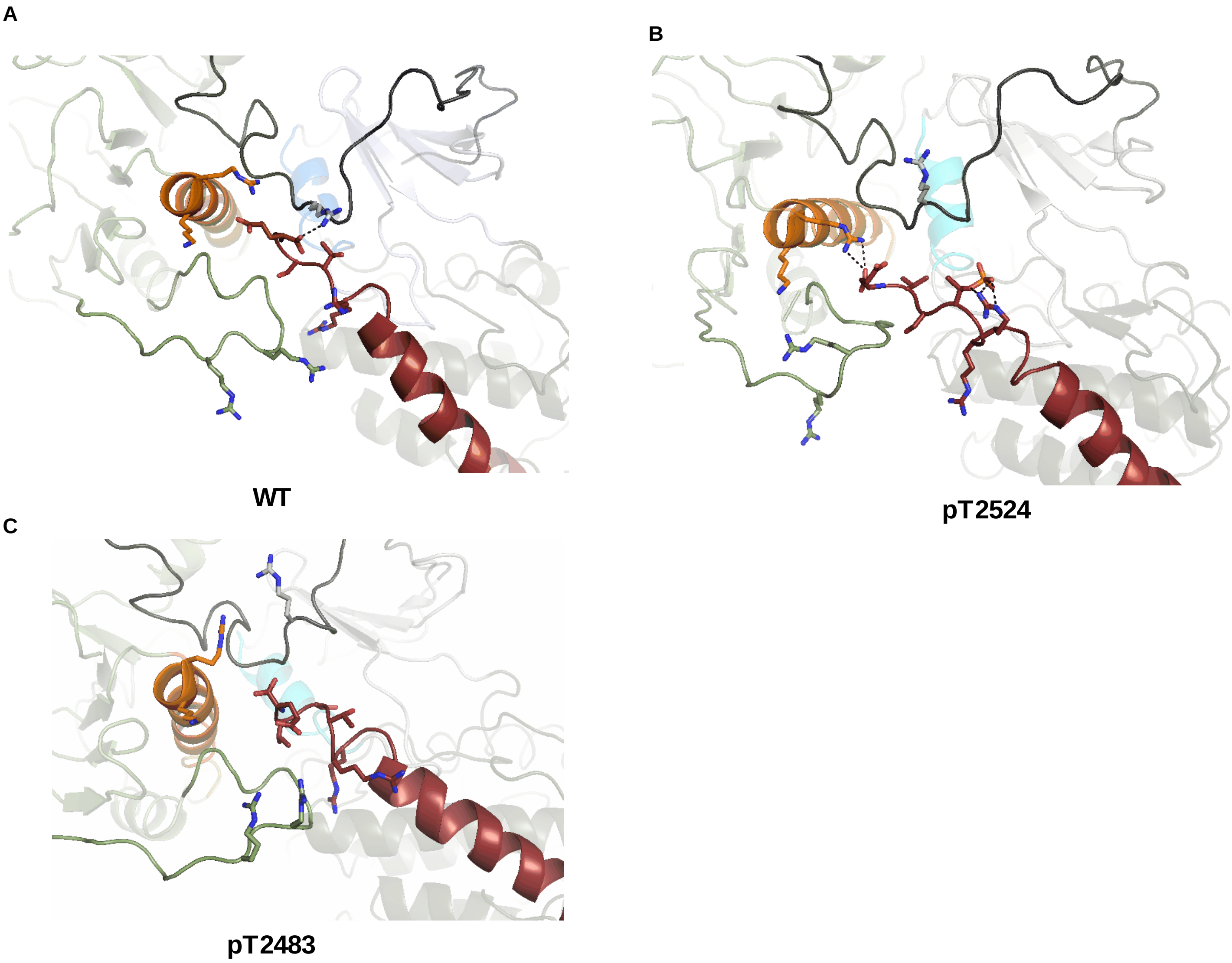

Supplementary movies. (A) Movie of wild-type RCKW highlighting the Ct helix, CORB helix, and CORB-kinase linker.

(B) Movie of wild-type RCKW phosphorylated at T2524 highlighting the Ct helix, CORB helix, and CORB-kinase linker.

(**C**) Movie of wild-type RCKW phosphorylated at T2483 highlighting the Ct helix, CORB helix, and CORB-kinase linker.
